# Identification of new participants in the rainbow trout (*Oncorhynchus mykiss*) oocyte maturation and ovulation processes using cDNA microarrays

**DOI:** 10.1186/1477-7827-4-39

**Published:** 2006-07-27

**Authors:** Julien Bobe, Jerôme Montfort, Thaovi Nguyen, Alexis Fostier

**Affiliations:** 1 Institut National de la Recherche Agronomique, INRA-SCRIBE, IFR 140, Campus de Beaulieu, 35000 Rennes Cedex, France

## Abstract

**Background:**

The hormonal control of oocyte maturation and ovulation as well as the molecular mechanisms of nuclear maturation have been thoroughly studied in fish. In contrast, the other molecular events occurring in the ovary during post-vitellogenesis have received far less attention.

**Methods:**

Nylon microarrays displaying 9152 rainbow trout cDNAs were hybridized using RNA samples originating from ovarian tissue collected during late vitellogenesis, post-vitellogenesis and oocyte maturation. Differentially expressed genes were identified using a statistical analysis. A supervised clustering analysis was performed using only differentially expressed genes in order to identify gene clusters exhibiting similar expression profiles. In addition, specific genes were selected and their preovulatory ovarian expression was analyzed using real-time PCR.

**Results:**

From the statistical analysis, 310 differentially expressed genes were identified. Among those genes, 90 were up-regulated at the time of oocyte maturation while 220 exhibited an opposite pattern. After clustering analysis, 90 clones belonging to 3 gene clusters exhibiting the most remarkable expression patterns were kept for further analysis. Using real-time PCR analysis, we observed a strong up-regulation of ion and water transport genes such as aquaporin 4 (aqp4) and pendrin (slc26). In addition, a dramatic up-regulation of vasotocin (avt) gene was observed. Furthermore, angiotensin-converting-enzyme 2 (ace2), coagulation factor V (cf5), adam 22, and the chemokine cxcl14 genes exhibited a sharp up-regulation at the time of oocyte maturation. Finally, ovarian aromatase (cyp19a1) exhibited a dramatic down-regulation over the post-vitellogenic period while a down-regulation of Cytidine monophosphate-N-acetylneuraminic acid hydroxylase (cmah) was observed at the time of oocyte maturation.

**Conclusion:**

We showed the over or under expression of more that 300 genes, most of them being previously unstudied or unknown in the fish preovulatory ovary. Our data confirmed the down-regulation of estrogen synthesis genes during the preovulatory period. In addition, the strong up-regulation of aqp4 and slc26 genes prior to ovulation suggests their participation in the oocyte hydration process occurring at that time. Furthermore, among the most up-regulated clones, several genes such as cxcl14, ace2, adam22, cf5 have pro-inflammatory, vasodilatory, proteolytics and coagulatory functions. The identity and expression patterns of those genes support the theory comparing ovulation to an inflammatory-like reaction.

## Background

In fish, as in other lower vertebrates, the post-vitellogenic period is very important for the completion of the oogenetic process. During this step, the follicle-enclosed post-vitellogenic oocyte undergoes several key events such as the final acquisition of the ability to resume meiosis in response to the maturation-inducing steroid (MIS), the resumption of the meiotic process and, finally, its release from the surrounding follicular layers. In addition, the whole follicle (oocyte and surrounding follicular cells) undergoes a progressive differentiation ultimately leading to the release of a metaphase 2 oocyte. The key hormonal and molecular events involved in the control of meiosis resumption have been thoroughly studied and many studies have been dedicated to the action of gonadotropins, the regulation of steroidogenenic events and the action of the MIS (see [1-6] for review). However, the associated follicular or extra-follicular events involved in concomitant processes such as oocyte-follicular cells cross talk and ovulationmechanisms have received far less attention. Nevertheless, several researchgroups have studied the periovulatory ovarian physiology using classical biochemical or histological tools and, later, molecular approaches. Thus, several studies have dealt with ovarian proteases in their participation in the ovulatory process [7-9]. Differential display PCR and suppressive subtractive hybridization (SSH) approaches have also been developed in order to identify new differentially regulated genes in the fish periovulatory ovary [10-13]. In addition, numerous candidate gene studies have also been performed in the fish periovulatory ovary. Apart from genes related to hormonal controls, these studies were mostly dedicated to some specific gene families such as TGF beta family [14,15] or connexins [16,17]. Finally, fewer studies have simultaneously analyzed the expression profiles of several genes belonging to different families [18,19]. However, in contrast to other biological processes, such as immune response [20], the post-vitellogenic period has never benefited from genome-wide transcriptomic studies that could provide a global view of the molecular events occurring in the post-vitellogenic ovary undergoing oocyte maturation. In this context, the present study aimed at performing a transcriptomic analysis of the post-vitellogenic rainbow (*Oncorhynchus mykiss*) trout ovary. In order to do so, 9152-gene rainbow trout cDNA microarrays were hybridized using RNA samples originating from rainbow trout ovarian tissue collected during late vitellogenesis, post-vitellogenesis and oocyte maturation. A statistical analysis was performed in order to identify all the genes exhibiting a differential expression over this period. In addition, a supervised clustering analysis was performed using only the differentially expressed genes in order to identify groups (or clusters) of genes exhibiting similar expression profiles. Furthermore, as a first step in a long-term transcriptomic analysis of the rainbow trout post-vitellogenic ovary, we deliberately chose to focus on 3 gene clusters exhibiting the most remarkable expression patterns. Finally, specific genes were selected in each cluster based on the novelty of their putative identity and/or function. For each gene, a real-time PCR analysis of their ovarian expression profiles was performed using additional ovarian RNA samples.

## Methods

### Animal and tissue collection

Investigations were conducted according to the guiding principles for the use and care of laboratory animals and in compliance with French and European regulations on animal welfare. Two year old female rainbow trout (*Oncorhynchus mykiss*) were obtained during their first reproductive season from our experimental fish farm (Sizun, France) and held under natural photoperiod in a re-circulated water system in INRA experimental facilities (Rennes, France). The water temperature was kept constant at 12°C. Ovaries were sampled from individual females during late vitellogenesis (N = 6), post-vitellogenesis (N = 6) and during oocyte maturation (N = 6). Oocyte developmental stage was assessed under binocular microscope according to previously described criteria [21,22]. Late vitellogenic samples were collected at the end of the vitellogenic process, approximately 3–4 weeks before expected ovulation. At this stage, germinal vesicle is not visible and no polarized cytoplasm area can be observed. Post-vitellogenic samples were collected 2–3 weeks later but before any noticeable morphological changes in yolk structure due to the process of meiosis resumption. At this stage, oocytes can display a subperipheral or peripheral germinal vesicle. When germinal vesicle is not visible, a dark mass of polarized cytoplasm can be observed. Oocyte maturation samples were collected after meiosis resumption. Those samples were thus collected after yolk clarification and around the time of germinal vesicle breakdown (GVBD). For tissue collection, trout were deeply anesthetized in 2-phenoxyethanol, killed by a blow on the head and bled by gill arch section. Ovaries were then dissected out of the body cavity under sterile conditions. Ovarian aliquots were frozen in liquid nitrogen and stored at -80°C until RNA extraction.

### RNA extraction and reverse transcription

Ovarian tissue was homogenized in Trizol reagent (Invitrogen, Cergy Pontoise, France) at a ratio of 100 mg per ml of reagent and total RNA was extracted according to manufacturer's instruction. Due to yolk abundance in rainbow trout full-grown oocytes, total RNA was subsequently re-purified using a Nucleospin RNA 2 kit (Macherey Nagel, Germany) to obtain genomic grade RNA quality.

### cDNA microarrays

Nylon micro-arrays (7.6 × 2.6 cm) were obtained from INRA-GADIE (Jouy-en-Josas, France) [23]. A set of 9152 distinct rainbow trout cDNA clones originating from a pooled-tissues library [24] were spotted in duplicates after PCR amplification. PCR products were spotted onto Hybond N+ membranes as described by Nguyen et al. [25]. This rainbow trout generic array was deposited in Gene Expression Omnibus (GEO) database (Platform# GPL 3650) [26].

### Hybridization

RNA samples originating from 13 ovarian samples (late vitellogenesis, N = 3; post-vitellogenesis, N = 4 and oocyte maturation N = 6) were used for microarray hybridization according to the following procedure. Hybridizations were carried out as described by Bertucci et al. [27], with minor modifications, at INRA AGENAE genomic facility (Rennes). A first hybridization was performed using a 33P-labelled oligonucleotide (TAATACGACTCACTATAGGG which is present at the extremity of each PCR product) to monitor the amount of cDNA in each spot. After stripping (3 hours 68°c, 0.1× SSC, 0.2% SDS), arrays were prehybridized for 1 h at 65°C in hybridization solution (5× Denhardt's, 5× SSC, 0.5% SDS). Complex probes were prepared from 3 μg of RNA of each sample by simultaneous reverse transcription and labeling for 1 hour at 42°C in the presence of 50 μCi [alpha-33P] dCTP, 5 μM dCTP, 0.8 mM each dATP, dTTP, dGTP and 200 units M-MLV SuperScript RNase H-reverse transcriptase (GIBCO BRL) in 30 μL final volume. RNA was degraded by treatment at 68°C for 30 min with 1 μl 10% SDS, 1 μl 0.5 M EDTA and 3 μl 3 M NaOH, and then equilibrated at room temperature for 15 min. Neutralization was done by adding 10 μl 1 M Tris-HCl plus 3 μl 2N HCl. Arrays were incubated with the corresponding denatured labeled cDNAs for 18 h at 65°C in hybridization solution. After 3 washes (1 hours 68°C, 0.1× SSC 0.2% SDS), arrays were exposed 65 hours to phosphor-imaging plates before scanning using a FUJI BAS 5000. Signal intensities were quantified using ArrayGauge software (FujifilmMedical Systems, Stanford, CT) and deposited in GEO database (Series# GSE 4871).

### Microarray signal processing

Low oligonucleotide signals (lower than three times the background level) were excluded from the analysis. After this filtering step, signal processing was performed using the vector oligonucleotide data to correct each spot signal by the actual amount of DNA present in each spot. After correction, signal was normalized by dividing each gene expression value by the median value of the array.

### Microarray data analysis

A statistical analysis was performed in order to identify differentially expressed genes between late vitellogenic, post-vitellogenic and maturing groups using SAM software[28]. Three 2-by-2 statistical analyses were performed in order to compare each group with the two other ones. In addition, a comparison was performed between samples taken prior to meiosis resumption (from late and post-vitellogenic females, N = 7) and during oocyte maturation (N = 6). For each comparison, the lowest false discovery rate (FDR) was used to identify differentially abundant genes. All genes identified in at least one of the above comparisons were kept for clustering analysis in order to characterize the expression profiles of statistically relevant genes. For supervised clustering analysis [29], data was log transformed, median-centered and an average linkage clustering was performed using CLUSTER software [29]. Clusters were visualized using TREEVIEW software [29].

### Data mining

Rainbow trout sequences originating from INRA Agenae [24] and USDA [30] EST sequencing programs were used to generate publicly available contigs [31]. The 8th version (Om.8, released January 2006) was used for BlastX [32] comparison against the Swiss-Prot database (January 2006) [33]. The score of each alignment was retrieved after performing a BlastX comparison. In addition, for each EST spotted onto the membrane, the accession number of the corresponding rainbow trout cluster (Unigene Trout, January 2006), if any, was retrieved from the UniGene database [34].

### Real-time PCR analysis

Real-time PCR was performed using all RNA (N = 18) samples including those used for microarray analysis. Several over and under expressed clones belonging to three selected remarkable clusters, were selected according to their putative identity and/or function for analysis. Reverse transcription and real time PCR were performed as previously described [19]. Briefly, 3 μg of total RNA were reverse transcribed using 200 units of Moloney murine Leukemia virus (MMLV) reverse transcriptase (Promega, Madison, WI) and 0.5 μg random hexamers (Promega) per μg of total RNA according to manufacturer's instruction. RNA and dNTPs were denatured for 6 min at 70°C, then chilled on ice for 5 min before the reverse transcription master mix was added. Reverse transcription was performed at 37°C for 1 hour and 15 min followed by a 15 min incubation step at 70°C. Control reactions were run without MMLV reverse transcriptase and used as negative controls in the real-time PCR study. Real-time PCR experiments were conducted using an I-Cycler IQ (Biorad, Hercules, CA). Reverse transcription products were diluted to 1/25, and 5 μl were used for each real-time PCR reaction. Triplicates were run for each RT product. Real-time PCR was performed using a real-time PCR kit provided with a SYBR Green fluorophore (Eurogentec, Belgium) according to the manufacturer's instructions and using 600 nM of each primer. After a 2 min incubation step at 50°C and a 10 min incubation step at 95°C, the amplification was performed using the following cycle: 95°C, 20 sec; 60°C, 1 min, 40 times. For all primer pairs, the relative abundance of target cDNA within sample set was calculated from a serially diluted ovarian cDNA pool using the I-Cycler IQ software. This dilution curve was used to ensure that PCR efficiency was within an 80–100% range and that amplification was linear within sample set. After amplification, a fusion curve was obtained using the following protocol: 10 sec holding followed by a 0.5°C increase, repeated 80 times and starting at 55°C. The level of 18S RNA in each sample was measured and used for target genes abundance normalization within sample set. In addition to the genes identified from the transcriptomic analysis, a widely used standard gene, elongation factor 1 alpha (*ef1*α), was monitored using the same sample set to validate the normalization procedure. GenBank accession number and primer sequences are shown in table 1. Statistical analyses were performed using Statistica 7.0 software (Statsoft, Tulsa, OK). Differences between ovarian developments stages were analyzed using non parametric U tests.

**Table 1:** Primer used for the real-time PCR study. For each target gene, full and abbreviated names, GenBank accession number of the corresponding rainbow trout sequence and primer sequences are shown. The clone # is consistent with clone numbering in Figure 1 and Tables 2–4.

**Target gene**	**Abbreviated name**	**GenBank #**	**Clone #**	**Forward sequence**	**Reverse sequence**
ovarian aromatase	*cyp19a1*	BX083177	196 198	CTCTCCTCTCATACCTCAGGTT	AGAGGAACTGCTGAGTATGAAT
vitamin K dependent protein S precursor	*protein S*	BX320624	199 200	ACATGTGGGGGATGTTCATT	GAGGCCATGTTACGGTTTTG
Cytidine monophosphate-N-acetylneuraminic acid hydroxylase	*cmah*	BX878414	212	GGAGGCCTGTTCATCAAAGA	CCTGTGTGAAGCTGTCAGGA
coagulation factor V	*cf5*	BX879767	235	AGGGACACACACACACATCC	GAGTTACTGCACGCACCTGA
pendrin or solute carrier family 26	*slc26*	BX873066	236	CATGCATGGATTCATGGAATAA	TGGATTGGGTGACATCAACA
vasotocin	*avt*	CA375992	238	GAGGCTGGAGGAAGAGTGTG	TTCTGTTTGCTGGGTGACTG
angiotensin-converting enzyme 2	*ace2*	BX867294	245	AACAACAGGAAGCCAGGATG	CGTTCCACATGTATGCCTTG
CXC chemokine L14	*cxcl14*	BX868653	250	CAAAGGGAACGAGTGAGAGAA	GCCTGATGGCCAACTTAAAC
A Disintegrin And Metalloproteinases 22	*adam22*	CA363158	258	CCCGACTAGGAGAGTTGCAG	ATCATCACATGACCCCCACT
serine protease 23	*sp23*	BX087643	296	ACTGCCGAGAAGGATGAAGA	CCTCAGCAAGGGAAGTGAAG
aquaporin 4	*aqp4*	BX885214	305 306	TGTCATTACCAGCCAACTGC	TGAGACAGCCCTCCAGAAGT
elongation factor 1 alpha	*ef1*α	AF498320		AGCGCAATCAGCCTGAGAGGTA	GCTGGACAAGCTGAAGGCTGAG
18S ribosomal RNA	*18S*	AF308535		CGGAGGTTCGAAGACGATCA	TCGCTAGTTGGCATCGTTTAT

## Results

### Statistical analysis and supervised clustering

**Figure 1:**
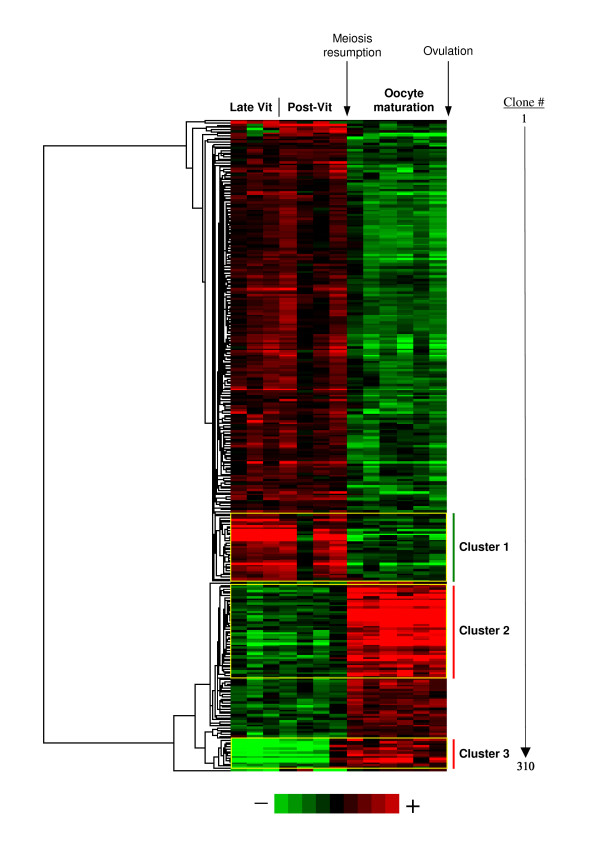
Supervised average linkage clustering analysis of 310 genes in the rainbow trout ovary during late vitellogenesis (Late Vit), post vitellogenesis (post-Vit) and oocyte maturation. Each row represents a gene and each column represents an ovarian RNA sample. The dendrogram on the left represents correlation distances between the profiles of studied genes. The 17 samples are supervised according to the natural time-course of oogenesis. For each gene the expression level within sample set is indicated using a color intensity scale. Red and green are used for over and under expression respectively while black is used for median expression.

After signal processing, 8263 clones out of 9152 were kept for further analysis. From the statistical analysis, 310 clones were found to exhibit a differential abundance between at least 2 of the studied ovarian stages (late vitellogenesis, post-vitellogenesis and oocyte maturation). For all SAM analyses performed, the false discovery rate (FDR) was always lower than 0.7%. Among the 310 identified clones, 90 were up-regulated during oocyte maturation while 220 exhibited an opposite pattern. A clustering analysis was performed using only expression data of the 310 identified clones in order to characterize the expression profiles of those genes. The clustering analysis clearly separated the over from the under expressed genes (Figure 1). The number of each clone (1–310) in the clustering analysis (Figure 1) was kept in subsequent tables 1, 2, 3, 4 and in the text. Within down-regulated genes, a cluster of 32 genes (cluster 1, Figure 1) was characterized by high expression levels during late vitellogenesis, low levels during oocyte maturation and intermediate or variable levels during post-vitellogenesis (Figure 1). Within up-regulated clones, a cluster of 44 genes (cluster 2, Figure 1) was characterized by a strong over expression at the time of meiosis resumption while a cluster of 14 genes (cluster 3, Figure 1) exhibited a very low expression during late and post-vitellogenesis and an up-regulation before meiosis resumption (Figure 1).

**Table 2:** Differentially regulated clones belonging to cluster 1.

**Clone name**	**#**	**GenBank**	**Sigenae contig**	**Swissprot_hit_description**	**Score**	**Unigene**
tcac0002.b.13	189	BX081818	tcac0002c.b.13_3.1.s.om.8	YBOX1_RAT (P62961) Nuclease sensitive element binding protein 1 (Y-box binding protein 1) (YB-1)	528	Omy.6894
tcay0023.c.11	190	BX311374	tcav0002c.e.18_3.1.s.om.8	IF2B_HUMAN (P20042) Eukaryotic translation initiation factor 2 subunit 2 (eIF-2-beta)	1096	Omy.8419
tcay0023.a.19	191	BX310198	tcay0023b.a.19_3.1.s.om.8 tcay0002b.h.16_3.1.s.om.8	ST1S3_BRARE (Q7T2V2) Cytosolic sulfotransferase 3 (EC 2.8.2.-) (SULT1 ST3)	1267	Omy.9054
tcba0022.f.09	192	BX868083	tcay0021b.l.08_3.1.s.om.8	NCPR_SALTR (P19618) NADPH – cytochrome P450 reductase (EC 1.6.2.4) (CPR) (P450R) (Fragments)	1536	Omy.22976
tcay0017.l.02	193	BX307506	tcay0017b.l.02_3.1.s.om.8 tcay0017b.l.02_5.1.s.om.8	NIPM_HUMAN (O43920) NADH-ubiquinone oxidoreductase 15 kDa subunit (EC 1.6.5.3) (EC 1.6.99.3)	382	Omy.3888
tcac0006.f.17	194	BX085016	tcac0006c.f.17_5.1.s.om.8 tcac0001c.e.23_3.1.s.om.8	CP2J3_RAT (P51590) Cytochrome P450 2J3 (EC 1.14.14.1) (CYPIIJ3)	712	Omy.18165
tcba0023.m.01	195	BX867932	tcay0010b.o.13_3.1.s.om.8	EXOS3_HUMAN (Q9NQT5) Exosome complex exonuclease RRP40 (EC 3.1.13)	668	Omy.7234
tcac0004.f.21	196	BX083177	tcac0004c.f.21_5.1.s.om.8 tcac0004c.f.21_3.1.s.om.8	CP19A_ORYLA (Q92087) Cytochrome P450 19A1 (EC 1.14.14.1) (Aromatase) (CYPXIX) (Estrogen synthetase) (P-450AROM)	879	Omy.241
tcac0002.f.11	197	BX081889	tcac0002c.f.11_3.1.s.om.8	RNPC2_HUMAN (Q14498) RNA-binding region containing protein 2 (Hepatocellular carcinoma protein 1) (Splicing factor HCC1)	1708	Omy.1045
tcbk0013.n.16	198	BX876154	tcac0004c.f.21_3.1.s.om.8	CP19A_ORYLA (Q92087) Cytochrome P450 19A1 (EC 1.14.14.1) (Aromatase) (CYPXIX) (Estrogen synthetase) (P-450AROM)	879	Omy.241
tcbk0006.j.01	199	BX874921	tcav0003c.p.16_5.1.s.om.8	PROS_BOVIN (P07224) Vitamin K-dependent protein S precursor	415	Omy.4204
tcay0036.n.19	200	BX320625	tcav0003c.p.16_3.1.s.om.8 tcav0003c.p.16_5.1.s.om.8	PROS_BOVIN (P07224) Vitamin K-dependent protein S precursor	415	Omy.4204
tcba0006.l.19	201	BX860777	tcay0018b.i.17_3.1.s.om.8	TFR1_CRIGR (Q07891) Transferrin receptor protein 1 (TfR1) (TR) (TfR) (Trfr)	968	Omy.16719
tcag0002.n.03	202	CT962587	tcag0002b.n.03_5.1.s.om.8	CP1A3_ONCMY (Q92109) Cytochrome P450 1A3 (EC 1.14.14.1) (CYP1A3) (CYP1A1)	2563	Omy.11738
tcab0003.h.21	203	BX080053	tcab0003c.h.21_5.1.s.om.8	RT30_MOUSE (Q9D0G0) Mitochondrial 28S ribosomal protein S30 (S30mt) (MRP-S30)	104	Omy.16941
tcak0001.o.11	204		tcaa0001c.e.22_5.1.s.om.8	RL4A_XENLA (P08429) 60S ribosomal protein L4-A (L1A)	1161	Omy.806
tcbk0003.k.18	205	BX874857	tcay0003b.p.21_3.1.s.om.8	RDH3_RAT (P50169) Retinol dehydrogenase 3 (EC 1.1.1.105) (Retinol dehydrogenase type I) (RODH I)	834	Omy.2974
tcay0037.m.03	206	BX319609	tcay0010b.o.11_3.1.s.om.8	GCST_MOUSE (Q8CFA2) Aminomethyltransferase, mitochondrial precursor (EC 2.1.2.10) (Glycine cleavage system T protein) (GCVT)	1289	Omy.6341
1RT64O23_A_H12	207	CA358010	tcac0005c.k.07_3.1.s.om.8	KAD2_BOVIN (P08166) Adenylate kinase isoenzyme 2, mitochondrial (EC 2.7.4.3) (ATP-AMP transphosphorylase)	979	Omy.10546
tcbk0003.b.17	208	BX873257	tcay0016b.b.23_3.1.s.om.8	UNKNOWN		Omy.8692
tcay0009.k.05	209	BX302690	tcay0009b.k.05_3.1.s.om.8 tcay0009b.k.05_5.1.s.om.8	HM13_MOUSE (Q9D8V0) Minor histocompatibility antigen H13 (EC 3.4.99.-) (Signal peptide peptidase) (Presenilin-like protein 3)	1015	Omy.24131
1RT36F13_B_C07	210	CA376488	tcay0002b.c.06_3.1.s.om.8	TCPQ_PONPY (Q5RAP1) T-complex protein 1, theta subunit (TCP-1-theta) (CCT-theta)	2366	Omy.9154
tcaa0001.g.20	211	BX073727	tcaa0001c.g.20_3.1.s.om.8 tcaa0001c.g.20_5.1.s.om.8	TPM4_PIG (P67937) Tropomyosin alpha 4 chain (Tropomyosin 4)	744	Omy.20509
tcbk0034.l.08	212	BX878414	tcbk0003c.j.07_5.1.s.om.8	CMAH_BRARE (Q6GML1) Cytidine monophosphate-N-acetylneuraminic acid hydroxylase (EC 1.14.18.2)	2024	Omy.4470
tcay0031.j.13	213	BX316758	tcaa0002c.f.05_3.1.s.om.8	TPM4_PIG (P67937) Tropomyosin alpha 4 chain (Tropomyosin 4)	772	Omy.8952
tcbk0045.m.11	214	BX884217	tcbk0028c.k.18_5.1.s.om.8	GSTP1_CRIMI (P47954) Glutathione S-transferase P (EC 2.5.1.18) (GST class-pi)	636	Omy.20977
tcbk0037.f.02	215	BX889865	tcbi0036c.o.04_5.1.s.om.8	AT11B_HUMAN (Q9Y2G3) Probable phospholipid-transporting ATPase IF (EC 3.6.3.1)	1193	Omy.18989
1RT146H05_B_D03	216	CA350003	tcbk0038c.p.05_5.1.s.om.8	HSP47_CHICK (P13731) 47 kDa heat shock protein precursor	904	Omy.24697
1RT138M15_A_G08	217	CA386530		UNKNOWN		
1RT129O11_A_H06	218	CA385378	CA385378.1.s.om.8	JPH2_HUMAN (Q9BR39) Junctophilin-2 (Junctophilin type 2) (JP-2)	554	
1RT148P13_B_H07	219	CA368032	CA350754.1.s.om.8	CI010_HUMAN (Q9NZB2) Protein C9orf10	103	
tcbk0039.a.06	220	BX886884	tcbk0007c.f.07_5.1.s.om.8	MDP1_PIG (P22412) Microsomal dipeptidase precursor (EC 3.4.13.19) (MDP) (Dehydropeptidase-I) (Renal dipeptidase) (RDP)	1230	Omy.21182

**Table 3:** Differentially regulated clones belonging to cluster 2

**Clone name**	**#**	**GenBank**	**Sigenae Contig**	**swissprot_hit_description**	**Score**	**Unigene**
1RT85J04_D_E02	222	CA345139	CA345139.1.s.om.8	UNKNOWN		
1RT62P05_B_H03	223	CA352834	CA352834.1.s.om.8	TIM14_BRARE (Q6PBT7) Mitochondrial import inner membrane translocase subunit TIM14 (DnaJ homolog subfamily C member 19)	525	Omy.10044
1RT124G08_C_D04	224	CA359690	tcay0005b.b.16_3.1.s.om.8	CITE2_HUMAN (Q99967) Cbp/p300-interacting transactivator 2 (MSG-related protein 1) (MRG1 protein) (P35srj)	208	Omy.6626
tcay0013.c.09	225	BX305023	tcay0013b.c.09_3.1.s.om.8	CALD1_HUMAN (Q05682) Caldesmon (CDM)	298	Omy.9824
1RT110O02_C_H01	226	CA366638	CA366638.1.s.om.8	FTHFD_PONPY (Q5RFM9) 10-formyltetrahydrofolate dehydrogenase (EC 1.5.1.6) (10-FTHFDH) (Aldehyde dehydrogenase 1 family member L1)	755	
tcad0009.n.15	227	BX081106	tcad0009a.n.15_3.1.s.om.8	GPX4_PIG (P36968) Phospholipid hydroperoxide glutathione peroxidase, mitochondrial precursor (EC 1.11.1.12) (PHGPx) (GPX-4)	633	Omy.18352
tcac0005.m.05	228	BX083339	tcac0005c.m.05_3.1.s.om.8	CP8B1_MOUSE (O88962) Cytochrome P450 8B1 (EC 1.14.-.-) (CYPVIIIB1)	466	Omy.1855
tcay0037.g.24	229	BX320606	tcay0037b.g.24_3.1.s.om.8	NOE2_HUMAN (O95897) Noelin-2 precursor (Olfactomedin-2)	571	Omy.278
1RT148F22_D_C11	230	CA368141	CA368141.1.s.om.8	ETS2_CHICK (P10157) C-ETS-2 protein	654	
1RT41I23_A_E12	231	CA376743	CA376743.1.s.om.8	UNKNOWN		
tcbk0013.j.22	232	BX872432	tcbk0006c.l.19_5.1.s.om.8	GPC3_HUMAN (P51654) Glypican-3 precursor (GTR2-2) (MXR7)	300	Omy.25417
tcba0030.f.01	233	BX865931	tcay0001b.n.04_3.1.s.om.8	BASI_HUMAN (P35613) Basigin precursor (CD147 antigen) (Leukocyte activation antigen M6) (Collagenase stimulatory factor)	427	Omy.19589
1RT164G02_C_D01	234	CA387850	tcbk0061c.m.06_5.1.s.om.8	SGK2_HUMAN (Q9HBY8) Serine/threonine-protein kinase Sgk2 (EC 2.7.1.37) (Serum/glucocorticoid regulated kinase 2)	1247	Omy.9898
tcbk0057.a.03	235	BX879767	tcba0016c.m.19_5.1.s.om.8	FA5_BOVIN (Q28107) Coagulation factor V precursor (Activated protein C cofactor)	832	Omy.16361
tcbk0013.j.13	236	BX873066	tcbk0013c.j.13_5.1.s.om.8	PEND_HUMAN (O43511) Pendrin (Sodium-independent chloride/iodide transporter) (Solute carrier family 26 member 4)	521	
1RT38L12_D_F06	237	CA377239	CA377239.1.s.om.8	DMD_CANFA (O97592) Dystrophin	660	
1RT34L03_B_F02	238	CA375992	tcai0003a.h.04_5.1.s.om.8	NEU3_ONCKE (P16041) Vasotocin-neurophysin VT 1 precursor	716	Omy.12737
1RT113L09_B_F05	239	CA365239	tcbk0019c.d.02_5.1.s.om.8	NEU1_ONCKE (Q91166) Isoticin-neurophysin IT 1	875	Omy.13912
tcbk0048.p.10	240	BX884149	tcbk0048c.p.10_5.1.s.om.8	COLL4_MIMIV (Q5UPS7) Collagen-like protein 4	224	Omy.14306
tcbk0046.i.17	241	BX884287	tcbk0046c.i.17_5.1.s.om.8	COPT1_MOUSE (Q8K211) High-affinity copper uptake protein 1 (CTR1)	640	
tcbk0004.a.22	242	BX876662	tcbk0004c.a.22_5.1.s.om.8	RGS18_HUMAN (Q9NS28) Regulator of G-protein signaling 18 (RGS18)	467	Omy.15619
tcba0003.a.09	243	BX857105	tcav0003c.k.16_3.1.s.om.8	UNKNOWN		Omy.11100
tcba0030.e.12	244	BX866986	tcay0011b.j.07_5.1.s.om.8	RNF24_HUMAN (Q9Y225) RING finger protein 24	286	
tcba0024.c.13	245	BX867294	tcav0002c.k.18_3.1.s.om.8	ACE2_HUMAN (Q9BYF1) Angiotensin-converting enzyme 2 precursor (EC 3.4.17.-)	1058	Omy.5193
1RT105A23_A_A12	246	CA363171	tcad0009a.b.12_3.1.s.om.8	GA45B_HUMAN (O75293) Growth arrest and DNA-damage-inducible protein GADD45	563	Omy.24221
tcbk0035.k.02	247	BX885992	tcbk0021c.h.17_5.1.s.om.8	FOXO3_HUMAN (O43524) Forkhead box protein O3A	880	Omy.21283
1RT148E11_A_C06	248	CA367914	tcay0003b.j.08_3.1.s.om.8	FOXO3_HUMAN (O43524) Forkhead box protein O3A	692	Omy.25125
tcbk0048.o.16	249	BX885768	tcbk0048c.o.16_5.1.s.om.8	SMOO_HUMAN (P53814) Smoothelin	717	
tcba0028.m.20	250	BX868653	tcav0001c.p.02_3.1.s.om.8	SCYBE_HUMAN (O95715) Small inducible cytokine B14 precursor (CXCL14)	319	Omy.2735
tcbk0053.e.07	251	BX879710	tcbk0053c.e.07_5.1.s.om.8	LFC_TACTR (P28175) Limulus clotting factor C precursor (EC 3.4.21.84) (FC)	183	
tcba0018.p.09	252	BX864334	tcba0018c.p.09_5.1.s.om.8	SGK2_HUMAN (Q9HBY8) Serine/threonine-protein kinase Sgk2 (EC 2.7.1.37)	1407	Omy.16859
tcba0013.e.11	253	BX863135	tcba0013c.e.11_5.1.s.om.8	RAMP1_RAT (Q9JJ74) Receptor activity-modifying protein 1 precursor	400	
tcba0016.h.07	254	BX863955	tcay0023b.e.18_3.1.s.om.8	ACY3_HUMAN (Q96HD9) Aspartoacylase-2 (EC 3.5.1.15) (Aminoacylase-3) (ACY-3) (Acylase III) (Hepatitis C virus core-binding protein 1) (HCBP1)	695	Omy.12550
tcba0028.o.19	255	BX866157	tcay0013b.p.20_3.1.s.om.8	VISL1_RAT (P62762) Visinin-like protein 1 (VILIP)	967	Omy.19419
1RT106P06_D_H03	256	CA365853	tcba0005c.a.01_5.1.s.om.8	MAFB_RAT (P54842) Transcription factor MafB (MAF1)	210	Omy.24386
1RT98J03_B_E02	257	CA357072	tcay0038b.i.24_5.1.s.om.8	PNPH_BOVIN (P55859) Purine nucleoside phosphorylase (EC 2.4.2.1)	964	Omy.15824
1RT106O19_A_H10	258	CA363158	CA363158.1.s.om.8	ADA22_XENLA (O42596) ADAM 22 precursor (MDC11b) (MDC11.2)	889	
1RT62L08_D_F04	259	CA352881	tcac0002c.j.24_3.1.s.om.8	TPP1_CANFA (Q9XSB8) Tripeptidyl-peptidase I precursor (EC 3.4.14.9)	599	Omy.8262
1RT44O11_A_H06	260	CA379089	tcay0014b.n.20_3.1.s.om.8	PSD2_HUMAN (Q13200) 26S proteasome non-ATPase regulatory subunit 2 (Tumor necrosis factor type 1 receptor associated protein 2) (55.11 protein)	1152	Omy.15261
1RT30D15_B_B08	261	CA372310	CA372310.1.s.om.8	KPCD_CANFA (Q5PU49) Protein kinase C, delta type (EC 2.7.1.-) (nPKC-delta)	932	
tcbk0050.j.02	262	BX890245	tcbk0050c.j.02_5.1.s.om.8	DMD_HUMAN (P11532) Dystrophin	1196	
1RT31H12_D_D06	263	CA375388	tcay0024b.g.05_3.1.s.om.8	UNKNOWN		Omy.4071
tcba0014.c.14	264	BX863437	tcav0005c.h.17_3.1.s.om.8	ELOV1_MOUSE (Q9JLJ5) Elongation of very long chain fatty acids protein 1	187	Omy.22915
tcad0006.j.09	265	BX077787	tcad0006a.j.09_5.1.s.om.8 tcad0006a.j.09_3.1.s.om.8	UNKNOWN		Omy.10230

**Table 4:** Differentially regulated clones belonging to cluster 3.

**Clone name**	**#**	**GenBank**	**Sigenae contig**	**swissprot_hit_description**	**Score**	**Unigene**
tcav0003.l.01	296	BX087643	tcav0003c.l.01_3.1.s.om.8 tcav0003c.l.01_5.1.s.om.8	PRS23_MOUSE (Q9D6X6) Serine protease 23 precursor (EC 3.4.21.-)	265	Omy.8589
tcbk0008.n.08	297	BX871436	tcac0002c.a.01_3.1.s.om.8	UNKNOWN		Omy.10950
tcad0003.m.13	298	BX075335	tcad0003a.m.13_5.1.s.om.8 tcad0003a.m.13_3.1.s.om.8	PPT1_MACFA (Q8HXW6) Palmitoyl-protein thioesterase 1 precursor (EC 3.1.2.22)	1110	Omy.3717
1RT65F10_D_C05	299	CA353171	tcab0001c.m.15_5.1.s.om.8	APOC1_MOUSE (P34928) Apolipoprotein C-I precursor (Apo-CI) (ApoC-I)	123	Omy.20585
tcay0008.f.19	300	BX301535	tcay0008b.f.19_3.1.s.om.8 tcay0008b.f.19_5.1.s.om.8	CLD11_MOUSE (Q60771) Claudin-11 (Oligodendrocyte transmembrane protein)	331	Omy.5138
1RT63M21_A_G11	301	CA357931	tcaa0002c.j.15_3.1.s.om.8	ION3_CARAU (P18520) Intermediate filament protein ON3	1331	Omy.40
1RT67D22_D_B11	302	CA360891	CA360891.1.s.om.8	PTPRF_HUMAN (P10586) Receptor-type tyrosine-protein phosphatase F precursor (EC 3.1.3.48) (LAR protein) (Leukocyte antigen related)	1466	Omy.24653
1RT63G21_A_D11	303	CA357905	tcad0003a.m.13_3.1.s.om.8	PPT1_MACFA (Q8HXW6) Palmitoyl-protein thioesterase 1 precursor (EC 3.1.2.22)	1110	Omy.5643
1RT35E10_C_C05	304	CA376275	CA376275.1.s.om.8	UNKNOWN		
tcbk0056.f.03	305	BX880542	tcbk0056c.f.03_5.1.s.om.8	AQP4_RAT (P47863) Aquaporin-4 (AQP-4) (WCH4) (Mercurial-insensitive water channel)	442	Omy.23866
tcbk0036.e.03	306	BX885214	tcbk0036c.e.03_5.1.s.om.8	AQP4_HUMAN (P55087) Aquaporin-4 (AQP-4) (WCH4) (Mercurial-insensitive water channel)	1071	Omy.23866
tcay0007.b.05	307	BX300900	tcay0007b.b.05_3.1.s.om.8	HEPH_RAT (Q920H8) Hephaestin precursor	1085	Omy.25044
tcac0006.o.01	308	BX085175	tcac0006c.o.01_3.1.s.om.8 tcac0006c.o.01_5.1.s.om.8	LTBP2_MOUSE (O08999) Latent transforming growth factor-beta-binding protein 2 precursor	328	
tcbk0044.e.02	309	BX889077	tcay0040b.e.18_5.1.s.om.8	UNKNOWN		Omy.23994

### Identity of differentially expressed cDNAs

The rainbow trout (*Oncorhynchus mykiss*) genome has not been sequenced and the number of characterized rainbow trout proteins and mRNAs is limited. The identity of studied transcripts was therefore based on the most significant hit obtained after performing a BlastX search against the SwissProt database. For the clones belonging to cluster 1–3, the results of this blast search is presented in tables 2, 3, 4. For each clone, the identity of the best hit in SwissProt and the score value of the BlastX comparison are given. However, this similarity search was performed using all EST sequences available in public databases and not using fully characterized cDNAs displaying the full coding sequence of the transcript. For some of the clones spotted on the trout array, the corresponding mRNA was previously characterized and made available in public databases. The identity of those clones is therefore unambiguous. In contrast, for some other clones, the best hit in SwissProt only gives significant, but incomplete, information. This is especially true for protein family members for which only a phylogenetic analysis will allow a more relevant identification of the gene. However, the name of the best hit was used in the text for clarity reasons.

#### Cluster 1

This large cluster of 32 clones (# 189–220) was characterized by a clear under expression at the time of oocyte maturation. Among those 32 clones, 29 belonged to a UniGene cluster and 30 had a significant hit in Swiss-Prot (Table 2). Two clones (# 196 and 198) corresponded to rainbow trout ovarian P450 aromatase (*cyp19a1*) and therefore belonged to the same UniGene cluster (Omy. 241). Similarly, clones # 199 and 200 belonged to UniGene cluster Omy.4204 and exhibited sequence similarity with bovine vitamin K-dependent protein S precursor. In addition, one clone (# 202) corresponding to rainbow trout *cyp1a3 *(EC 1.14.14.1), was identified while another clone (# 194) was most similar to rat CYP2J3. Finally, this cluster also included clones exhibiting sequence similarity with zebrafish cytidine monophosphate-N-acetylneuraminic acid hydroxylase (*cmah*) (# 212), salmon NADPH – cytochrome P450 reductase (# 192) and Glutathione S-transferase (# 214). Within cluster 1, *cyp19a1 *(clones # 196 and 198), vitamin K-dependent protein S precursor (clones # 199 and 200) and *cmah *(clone # 212) genes were kept for real-time PCR analysis.

#### Cluster 2

This very large cluster of 44 clones (# 222–265) was characterized by a sharp over expression at the time of meiosis resumption. Among the 44 clones present in this cluster, 30 belonged to a UniGene cluster (Table 3). In addition, 39 clones exhibited a significant hit in SwissProt while 5 clones had no significant sequence similarities with known genes (Table 3). Within this cluster, several genes exhibited inflammation or ovulation-related functions. Thus some of the clones exhibited sequence similarities with human chemokine *cxcl14 *(clone # 250), clawed frog *adam22 *(clone # 258) and coagulation factor V (*cf5*) (clone # 235). In addition, one clone (# 245) exhibited strong sequence similarity with human angiotensin-converting enzyme 2 precursor (*ace2*). Two clones (# 238 and 239) exhibited strong sequence similarity with salmon (*Oncorhynchus keta*) vasotocin-neurophysin (*avt*) and isotocin-neurophysin respectively. Finally, cluster 3 also contained clones exhibiting sequence similarity with, human Forkhead box protein O3A and human pendrin, also know as solute carrier family 26 member 4 (*slc26*) (clone # 236). Within cluster 2, *cxcl14*, *adam22*, *slc26, avt, ace2 *and *cf5 *genes were kept for real-time PCR analysis.

#### Cluster 3

This small cluster of 14 clones (# 296–309) was characterized by an over expression occurring earlier than for the genes belonging to cluster 3. Among those 14 clones, 12 belonged to a UniGene cluster and 11 had a significant hit in SwissProt (Table 4). Two clones (# 305 and 306) were most similar to rat and human aquaporin 4 (*aqp4*) respectively. These 2 clones belonged to the same UniGene cluster (Omy.23866). In addition, one clone (# 296) was most similar to mouse serine protease 23 (*sp23*). Within cluster 3, *aqp4 *and *sp23 *genes were kept for real-time PCR analysis.

### Real-time PCR analysis

For all the genes selected for the real-time PCR analysis, a similar up or down regulation was observed between microarray and real-time PCR experiments.

#### Under expressed genes during oocyte maturation

**Figure 2:**
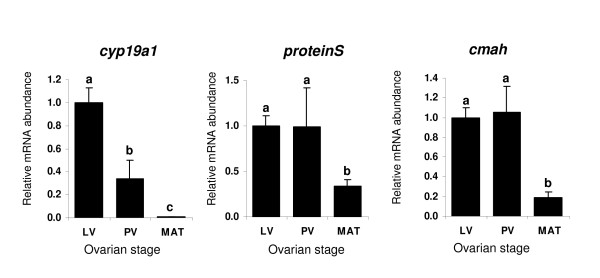
Ovarian expression profiles of aromatase (*cyp19a1*), vitamin K dependent protein S (*proteinS*) and cytidine monophosphate-N-acetylneuraminic acid hydroxylase (*cmah*) genes during rainbow trout late oogenesis (mean ± SEM). Ovaries were sampled from separate females during late vitellogenesis (**LV**, N = 6), post-vitellogenesis (**PV**, N = 6) and oocyte maturation (**MAT**, N = 6). The mRNA abundance of each gene was determined by real-time PCR and normalized to the abundance of 18S. Abundance was arbitrarily set to 1 for LV stage and data are expressed as a percentage of the transcript abundance at this stage. Bars sharing the same letter(s) are not significantly different (p < 0.05).

We observed a dramatic under expression of aromatase (*cyp19a1*, clones # 196 and 198) in the ovary during the preovulatory period (Figure 2). The mRNA abundance of *cyp19a *gene during oocyte maturation was more than 200 times lower than during late vitellogenesis. In addition, successive decreases of *cyp19a *gene expression levels were observed during post-vitellogenesis and during oocyte maturation (Figure 2). The mRNA abundance of vitamin K-dependent protein S precursor gene (clones # 199 and 200) was lower during oocyte maturation than during late or post-vitellogenesis. In contrast, no significant differences were observed between late and post-vitellogenesis (Figure 2). A similar expression profile was observed for Cytidine monophosphate-N-acetylneuraminic acid hydroxylase (*cmah*) gene (Figure 2).

#### Over expressed genes during oocyte maturation

**Figure 3:**
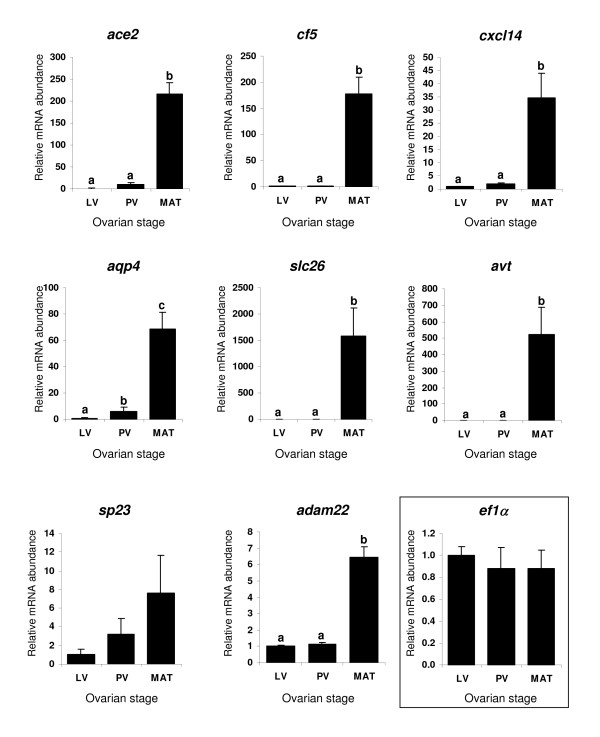
Ovarian expression profiles of angiotensin-converting enzyme 2 (*ace2*), coagulation factor V (*cf5*), CXC chemokine L14 (*cxcl14*), aquaporin 4 (*aqp4*), pendrin (*slc26*), vasotocin (*avt*), serine protease 23 (*sp23*), ADAM22 (*adam22*), and elongation factor 1 alpha (*ef1*α) genes during rainbow trout late oogenesis (mean ± SEM). Ovaries were sampled from separate females during late vitellogenesis (**LV**, N = 6), post-vitellogenesis (**PV**, N = 6) and oocyte maturation (**MAT**, N = 6). The mRNA abundance of each gene was determined by real-time PCR and normalized to the abundance of 18S. Abundance was arbitrarily set to 1 for LV stage and data are expressed as a percentage of the transcript abundance at this stage. Bars sharing the same letter(s) are not significantly different (p < 0.05).

We observed a strong over expression of aquaporin 4 (*aqp4*) gene during post-vitellogenesis and at the time of oocyte maturation (Figure 3). The mRNA abundance of a*qp4 *gene exhibited a 6-fold increase during post-vitellogenesis and a further 12-fold increase during oocyte maturation. In addition, the mRNA abundance of pendrin (*slc26*) gene exhibited a 1500-fold increase during oocyte maturation while no significant differences were observed between late and post-vitellogenesis. Similarly, vasotocin (*avt*) mRNA abundance exhibited a 500-fold increase at the time of oocyte maturation (Figure 3). Angiotensin-converting enzyme 2 (*ace2*) gene expression levels exhibited a 215-fold increase between late vitellogenesis and oocyte maturation (Figure 3). A similar profile was observed for the chemokine *cxcl14 *gene. The mRNA abundance of this gene exhibited a 35-fold increase between late vitellogenesis and oocyte maturation (Figure 3). The mRNA abundance of coagulation factor V (*cf5*) gene exhibited a 177-fold increase between late or post-vitellogenesis and oocyte maturation while a*dam22 *mRNA abundance exhibited a 6-fold increase between late or post-vitellogenesis and oocyte maturation (Figure 3). Finally, the mRNA abundance serine protease 23 (*sp23*) gene monitored during oocyte maturation was higher than in the late vitellogenic ovary. However, this difference was not significantly different (p = 0.078).

#### Control gene

The mRNA abundance of elongation factor 1 alpha (*ef1*α), a translation regulatory protein commonly used as a stable reference, did not exhibit any significant difference over the preovulatory period (Figure 3).

## Discussion

### Microarray analysis efficiency and reliability

The hybridization of radiolabeled cDNAs with cDNAs deposited on nylon membranes has been used for several decades. However, the use of nylon cDNA microarrays is not very common in comparison to glass slide microarray technology. Nevertheless, this technology has successfully been used for several years [27,35]. In the present study we used similar cDNA manufacturing and hybridization protocols. While most of the 9152 clones used to generate the microarray putatively correspond to distinct genes, a small proportion of genes are represented by 2 distinct clones (e.g clones belonging to the same UniGene cluster). In our data, it is noteworthy that those clones are usually found in the same gene clusters (e.g clones #196 and 198, #199 and 200, #305 and 306). Since the position of clones in the clustering analysis is based on the correlation between their profiles, this indicates that they display very similar expression profiles. In addition, for all genes selected for real-time PCR analysis, the over or under expression observed was always consistent with microarray data. Furthermore, the expression of *ef1*α, a widely used reference gene, was stable over the preovulatory period. Together, these observations suggest that our overall microarray analysis is extremely robust and reliable.

### Identities of identified genes and putative involvement in preovulatory ovarian functions

In the present study, we identified 310 genes exhibiting a differential expression during the preovulatory period. Among them, 220 were down-regulated during oocyte maturation while 90 exhibited an opposite pattern. However, because we decided, as a first step, to focus our analysis on the genes exhibiting the most differential regulation in the periovulatory period, we only present the identity of the 90 genes belonging to 3 specific clusters exhibiting the most remarkable patterns. Among those 90 transcripts we have chosen to discuss the most informative or novel genes based on their identities and/or putative involvement in the rainbow trout preovulatory ovarian functions.

#### Estrogen synthesis

Among the 32 clones belonging to cluster 1, two clones correspond to rainbow trout ovarian aromatase (*cyp19a1*). The real-time PCR study confirmed that *cyp19a1 *was dramatically under expressed during the preovulatory period. This observation is in total agreement with existing data on aromatase expression during this period [19,36]. In addition, a clone putatively encoding for a NADPH-cytochrome P450 reductase (EC 1.6.2.4) was also located in cluster 1. The aromatase enzyme complex is formed from 2 principal protein components. CYP19a1 contains the catalytic domain that binds C19 steroid substrates in the proximity of the heme prosthetic group critical in the activation of molecular oxygen and subsequent substrate hydroxylation. The other essential component is the redox partner flavoprotein, NADPH cytochrome P450 reductase. Interestingly, present data show that both transcripts exhibited an under expression during the rainbow trout preovulatory period, although it should be confirmed that the identified clone is coding for the oxydoreductase protein involved in the aromatase complex.

#### Other cytochrome P450 genes

Two other cytochrome P450 genes, exhibiting similar expression profiles were found in the same cluster. One clone (# 194) was most similar to rat cytochrome P450 2J3 while the other one (# 202) putatively corresponded to rainbow trout cytochrome P450 1A3 (*cyp1a3*). Cytochrome P450 1A proteins are ubiquitous proteins that have been associated with the detoxification of several organic compounds such as PCB (polychlorinated biphenyl), PAH (polyaromatic hydrocarbons), and dioxin [37]. In fish, these compounds are able to induce *cyp1a *gene expression in a variety of tissues. In the rainbow trout immature ovary, a constitutive expression of CYP1A protein was previously reported [38]. Together, previous and present observations suggest that a CYP1A-related detoxification activity in the rainbow trout ovary. From the under expression of *cyp1a3 *gene observed in the ovary immediately prior to ovulation we could speculate that a decrease of the detoxification activity of the ovary is required before the beginning of the ovulation process. In addition, it was previously shown in rat C6 glioma cells that epoxygenases could inhibit prostaglandin E2 production [39]. Interestingly, C6 cells express epoxygenase mRNAs, CYP1A1, CYP2B1 and CYP2J3, which convert arachidonic acid to epoxyeicosatrienoic acids; those epoxyeicosatrienoic acid being able to inhibit the activity of cyclooxygenase [39]. The role of prostaglandins in the ovulatory process has been thoroughly studied (see [40] for review). Thus, in rainbow trout, prostaglandin F2α was able to induce in vitro ovulation [21,41]. Therefore, the observed down-regulation of *cyp1a1 *and *cyp2j3 *genes in the ovary prior to ovulation is therefore totally consistent with available data on the participation of prostaglandins in the ovulatory process.

#### Ion/water transport genes

In the present transcriptomic analysis, two aquaporin 4 (*aqp4*) clones were found in cluster 3. Real-time PCR data confirmed that rainbow trout *aqp4 *gene exhibited a strong over expression in the preovulatory ovary. In mammals, AQP4 is also known as mercurial insensitive water channel (MIWC). It was previously shown that water permeability was strongly increased in African clawed frog oocytes expressing MIWC [42]. In marine fish, a strong oocyte hydration occurs during oocyte maturation [43,44]. In addition, it was recently shown that this oocyte hydration involves an aquaporin1-like protein in seabream [45]. In freshwater species, data on oocyte hydration is more controversial. However, a limited but significant hydration was also observed in several freshwater species including rainbow trout [46]. Our data suggest that, similarly to marine species, the oocyte hydration occurring during oocyte maturation could also be aquaporin-mediated in freshwater species such as rainbow trout. In addition to *aqp4 *gene, we also observed a dramatic over expression of *slc26 *gene at the time of meiosis resumption. Solute carrier family 26 member 4 (*slc26*) is also known as sodium-independent chloride/iodide transporters or pendrin. The over expression of *slc26 *gene at the time of oocyte maturation is dramatic, as demonstrated by real-time PCR. Together, the strong up-regulation of *aqp4 *and *slc26 *genes at the time of meiosis resumption stresses the importance of water and ion transports in the rainbow trout preovulatory ovarian functions. In marine species, the major oocyte hydration occurring before ovulation is probably important for adjusting egg buoyancy. In contrast, in freshwater species laying demersal eggs such as rainbow trout, it has been hypothesized that the limited (25%) oocyte hydration occuring before ovulation could be necessary for the completion of the ovulation process [46]. Thus, the increase of oocyte volume could facilitate the rupture of the follicular walls and subsequently, the release of the oocyte from its follicular layers.

The neurophysial hormones arginine vasotocin (AVT) and isotocin (IT) are the fish counterparts of arginine-vasopressin and oxytocin respectively. Vasotocin precursor and isotocin precursor cDNAs were previously cloned in several fish species including chum salmon [47,48]. In fish, AVT is involved in several physiological processes including water conservation and excretion of electrolytes [49]. However, existing data in fish correspond to the local effect, in various tissues, of circulating AVT [49]. Surprisingly, we observed that AVT precursor (*avt*) mRNA is expressed in the rainbow trout preovulatory ovary. To the best of our knowledge, there is no evidence of non-neural expression of *avt *mRNA in fish. In addition, it is noteworthy that we also observed a similar over expression of isotocin mRNA precursor in the ovary at the time of oocyte maturation. Further investigations are needed to elucidate the role of AVT and IT in the trout preovulatory ovarian functions.

#### Inflammation- or ovulation-related genes

Ovulation is a complex process resulting in the release of the oocyte from surrounding follicular layers. Since the early eighties, the similarities between ovulatory and inflammatory processes have been thoroughly discussed [50-52] and it is now well accepted that mammalian ovulation is an inflammatory-like reaction. In fish, despite numerous studies on the hormonal control of spawning, the ovulatory process has been far less documented.

In mammals, ovulation is accompanied by broad-spectrum proteolysis and the implication of several classes of proteases is well documented (see [53] for review). In salmonid fish, several proteases have been identified in the periovulatory ovary [54]. In mammals, there is evidence that mature ovarian follicles contain proteolytic enzymes, including serine proteases. Indeed, serine proteases have been implicated in both ovulatory and inflammatory reactions (see [50] for review). In the present study, serine protease 23 (*sp23*) gene appears progressively up-regulated during the preovulatory period. To our knowledge, *sp23 *gene expression was never reported in the periovulatory ovary of any vertebrate species. However, we could speculate that this protease participates in the rainbow trout ovulatory process. Interestingly, our data showed that *adam22 *metalloprotease-disintegrin gene was sharply up-regulated at the time of oocyte maturation. The metalloprotease-disintegrin protein family (also known as ADAMs: A Disintegrin And Metalloproteinases) is thought to function in cell-cell interactions and in the proteolysis of luminal or extracellular protein domains. In mammals, several ADAMs family members are involved in the ovulatory process. In brook trout (*Salvelinus fontinalis*), metalloprotease activity increases in the ovary prior to ovulation [8,9]. Together, these observations also suggest that *adam22 *also participates in the rainbow trout ovulatory process.

Mammalian CXC chemokines, named after a conserved pattern of conserved cysteine residues, have been initially identified as potent mediators of neutrophil chemotaxis [55,56] and are also involved in chemotaxis of monocytes and lymphocytes. They have also been implicated in angiogenesis and, later, in a large variety of functions[57,58]. In mammals, 16 CXC have been described. In Fish, however, several CXC have been identified but only CXCL12 and CXCL14 exhibit unambiguous orthologues [59]. In the present study, we showed that *cxcl14 *gene expression strongly increases during the preovulatory period. In catfish, RT-PCR data showed that *cxcl14 *gene was expressed in a wide variety of tissues, including the ovary [60]. In carp, quantitative PCR data showed that *cxcl14 *was predominantly expressed in the brain [61]. Despite its good conservation throughout vertebrate evolution [59], the number of studies addressing the *in vivo *role(s) of CXCL14 is limited. As a consequence, a lot of information is still unavailable in fish. In a murine model used to study Crohn's disease, *cxcl14 *expression is induced during inflammation [62]. Together, these observations suggest that *cxcl14 *gene expression induction contributes to the inflammatory-like events occurring in the rainbow trout at the time of ovulation. To date the participation of this gene in preovulatory ovarian functions was unsuspected.

In mammals, coagulation factor V participates in the coagulation process. In zebrafish, a coagulation factor V (*cf5*) cDNA was previously characterized [63]. According to these authors, several lines of evidences including biochemical and phylogenetic analyses suggest that the modern coagulation pathways found in mammals could also be functional in fish. Furthermore, it was previously shown that cultured rabbit macrophages were able to generate factor V procoagulant activity [64]. In the present study, we observed a dramatic increase of *cf5 *gene expression in the ovary during oocyte maturation. However, no significant difference was observed between late and post-vitellogenesis. From these observations we could speculate that, immediately prior to ovulation, the trout ovary secretes coagulation factors in order to prevent bleeding from ruptured ovarian follicles at the time of ovulation. Interestingly, the transcriptomic analysis showed that a transcript exhibiting sequence similarity with clotting factor C (Clone # 251, Table 3) was also over expressed immediately prior to ovulation.

Angiotensin-converting enzyme (ACE) cleaves Angiotensin I (Ang I) to form Angiotensin II (Ang II). Angiotensin-converting enzyme 2 (ACE2) is a recently described ACE homolog [65]. Both ACE and ACE2 are zinc-dependent peptidases of the M2-metalloprotease family. Within the renin-angiotensin system (RAS), ACE2 competes with ACE because it is capable of hydrolyzing Ang I into the nonapeptide Ang(1–9) [65]. In humans, *ace2 *gene expression was predominantly detected by Northern blot analysis in kidney, heart and testis [65,66]. In addition, a moderate expression was also observed in several other tissues including the ovary [66]. Using semi-quantitative RT PCR, a wide distribution was observed in rat tissues [67]. In mammals, previous observations suggested that the renin-angiotensin system was functional in the ovary. In cattle, a greater expression of Ang II was observed in large follicles. In addition, several lines of evidence supported the idea of Ang II in blocking the inhibitory effect of theca cells on meiosis resumption of bovine oocytes [68]. In brook trout (*Salvelinus fontinalis*) salmon Ang I and human Ang II were both able to increase the level of *in vitro *spontaneous ovulation [69]. In the present transcriptomic study, we observed a dramatic increase of *ace2 *gene expression during the preovulatory period. This observation was confirmed by real-time PCR data. Together, these observations suggest that the dramatic up-regulation of *ace2 *gene immediately prior to ovulation is important for the ovulatory process. In mammals, little is know about the role of ACE2 in the ovary. However, it is known in mammals that ACE2 can function as an Ang II degrading enzyme, forming the vasodilatator peptide Ang(1–7) [70,71]. Interestingly, a local vasodilatation is a key characteristics of the inflammatory response that is also observed during the mammalian ovulatory process (see [50] for review). Therefore, it can be hypothesized that the observed increase of *ace2 *gene expression in the trout preovulatory participates in the vascular dynamics changes that are putatively occurring during the ovulatory process.

#### Genes involved in the synthesis of egg components

Cytidine monophosphate-N-acetylneuraminic acid hydroxylase (CMAH) is the key enzyme for the synthesis of *N*-glycolylneuraminic acid. In salmonid eggs, cortical alveoli contain polysialoglycoproteins (PSGP). In rainbow trout, it was previously shown that those PSGP contain *N*-glycolylneuraminic acid residues [72]. In the present study we observed a significant decrease of *cmah *gene expression at the time of oocyte maturation. While the presence of *cmah *gene expression in the ovary is totally consistent with the presence of *N*-glycolylneuraminic acid in rainbow trout cortical alveoli content, it seems however difficult to speculate on the dynamics of PSGP accumulation in the oocytes.

## Conclusion

Our observations further confirmed that a progressive shut down of estrogen synthesis genes expression occurs in the ovary prior to meiosis resumption. In addition to already well studied genes such as aromatase, the present work shows that other genes exhibit a similar down-regulation, thus suggesting their participation in the preovulatory decrease of circulating estrogen levels.

In addition, we observed a strong up-regulation of ion/water transport genes in the preovulatory ovary. The identity of those genes is consistent with the recent identification of aquaporin mediated mechanisms in the fish oocyte hydration process and further supports the recent description of a limited but significant oocyte hydration occurring in the rainbow trout preovulatory ovary.

Finally, in addition to oocyte hydration-related genes, we also observed a strong over expression of several genes such proinflammatory factors, coagulation/clotting factors, vasodilatation factors and proteases in the ovary immediately prior to ovulation. Together, these observations suggest that, similarly to the theory developed in mammals, fish ovulation could also be compared ton an inflammatory-like reaction. In addition, the identification of those genes will allow specific studies leading to a better understanding of the ovulatory process in fish.

In the future, a global analysis of differentially regulated genes, based on their ontologies, is needed to satisfyingly describe preovulatory ovarian mechanisms. In addition, a cellular localization of gene expression will contribute in the understanding of their respective roles in the preovulaory ovarian physiology. Nevertheless, the present study clearly demonstrates that distinct (i.e. steroidogenic, proteolytic, proinflammatory) but concomitant events occur in the preovulatory ovary. Together, all those events concur to achieve the same goal which is the release, at the time of ovulation, of a fully competent oocyte, ready to be fertilized.

## Authors' contributions

JM performed the microarray analysis. TN performed the real-time PCR analysis. AF participated in the writing of the manuscript and in the design and coordination of the study. JB supervised the study, participated in the microarray and real-time PCR analyses, performed data mining analysis and drafted the manuscript. All authors read and approved the final manuscript.
